# Systematic minireview of the craniocervical junction in dogs with and without brachycephaly

**DOI:** 10.3389/fvets.2024.1416670

**Published:** 2024-05-31

**Authors:** Lukas Wess, Sibylle Kneissl

**Affiliations:** Diagnostic Imaging, Department of Small Animals and Horses, University of Veterinary Medicine, Vienna, Austria

**Keywords:** craniocervical junction, occipital hypoplasia, atlantoaxial overlapping, syringohydromyelia, brachycephalic, toy breed dog

## Abstract

**Objective:**

To identify, quantify and compare clinical and concurrent imaging findings of occipital hypoplasia (OH), syringomyelia (SM) and atlanto-occipital overlapping (AO) in dogs with or without brachycephaly.

**Methods:**

A focused systematic search for literature was performed in the Web of Science™, PubMed and Google Scholar databases. Both authors screened and classified the identified articles using EndNote and appraised the articles using the Critical Appraisal Skills Program checklists. The main clinical and concurrent imaging features were extracted and evaluated for coexistence of OH, SM, AO, and other imaging findings.

**Results:**

Thirty-one articles were included in this minireview. For articles focusing on descriptions of OH, SM and AO, 249 dogs had at least one of these conditions, and 3 of these 249 dogs (1%) had coexistence of all three conditions. For articles focusing on descriptions of the dogs, OH, SM, and AO were identified in 552/19/11/11, 574/2/0/6, and 100/0/0/0 small brachycephalic, small non-brachycephalic, large brachycephalic, and large non-brachycephalic breeds, respectively. For all small brachycephalic dogs, the percentages of affected animals were 40% for OH (*p* = 0.01), 42% for SM (*p* < 0.01) and 7% for AO (*p* = 0.033). The number of dogs having AO and clinical symptoms is low (*n* = 5).

**Conclusion:**

OH, SM and AO are more likely to affect small dogs. AO might be limited to small brachycephalic breeds owing to the geometry of the craniocervical junction. Hence, AO alone might not lead to SM. In individual dogs, readers should carefully interpret the clinical relevance of OH or AO in the absence of SM.

## 1 Introduction

Although the inability to breathe normally is considered the greatest obstacle in the welfare of small brachycephalic dogs (1), neurological, ophthalmological, dermatological, thermoregulatory, and gastrointestinal problems are also common in brachycephalic breeds (2, 3). When focusing on the craniocervical junction, four morphological alterations have been described. First, an unusual early closure of spheno-occipital synchondrosis at 8–12 months of age (6 months earlier than reported in non-brachycephalic breeds) causes the typical head shape (4). Second, occipital dysplasia results in either a slightly increased dorsal notch or an unusually enlarged foramen magnum. Other names for occipital dysplasia are caudal occipital malformation syndrome and occipito-atlantoaxial malformation (5–7). Absence or shortening of parts of the occipital bone has also been described as incomplete ossification (8, 9). Third, incomplete ossification of the atlas has been described as predisposing to atlantoaxial subluxation (10, 11). Overall, changes in the conformation of bony structures in the craniocervical junction are associated with individual differences in head and neck movements (12). Fourth, occipital hypoplasia describes the shortening of the basilar part of the occipital bone, causing a reduced volume in the caudal fossa. This can lead to overcrowding and subsequently to herniation of cerebellar matter through the foramen magnum, resembling Chiari-like malformation in humans. In addition to congenital abnormalities, a frequently described acquired abnormality in this region is syringomyelia (SM), which is characterized by fluid-filled cavities in the spinal cord parenchyma. While the exact pathophysiology of SM is unknown, it is related to an outflow obstruction of cerebrospinal fluid at the level of the foramen magnum and is described secondary to OH. Clinical signs may be like those of Chiari-like malformation or may be clinically silent (13–15). Atlanto-occipital overlapping (AO) has been described as non-traumatic overlapping of the occipital bone and neural arch of the atlas (7, 16–18). Imaging findings include diminished distance between the dorsal lamina of the atlas and the supraoccipital bone, with the dorsal lamina of the atlas located either immediately caudal to the foramen magnum or within it (16, 18, 19). AO has been implicated in the development of neuropathic pain and neurological dysfunctions such as ataxia (20).

The main objective of this systematic review was to identify, quantify and compare concurrent major imaging findings of OH, SM and AO in dogs with or without brachycephaly. The specific aims were to document the prevalence of SM with OH or AO in brachycephalic and small breed dogs compared with non-brachycephalic and large breed dogs, to list corresponding reported clinical symptoms, and to reevaluate the clinical relevance of AO in small brachycephalic dogs.

## 2 Materials and methods (search)

This review was conducted in accordance with the Preferred Reporting Items for Systematic Reviews and Meta-Analyses (PRISMA) statement (21). A systematic search for literature concerning the craniocervical junction was performed in the Web of Science^™^, PubMed and Google Scholar databases. The following keywords were searched: “occipital hypoplasia” OR “occipital dysplasia” OR “foramen magnum dysplasia” OR “incomplete ossification” OR “atlantoaxial overlapping” OR “syringohydromyelia” OR “syringomyelia” OR “Chiari-like malformation” AND “dog.” Original articles, systematic reviews and meta-analyses were retrieved. The identified articles were imported to EndNote 21 [Clarivate Analytics (US) LLC, London, UK] and classified by both authors. Reasons for exclusions were duplicates or the article had a different focus, an undefinable population, or an impossible classification of corresponding imaging findings. Retrieved articles were appraised using the Critical Appraisal Skills Program (CASP) checklists for the quality and importance of their concepts, facts, and conclusions. To follow the aims of the study, the focus of review was (a) documentation of dogs having at least two reviewed imaging signs (including OH, SM, or AO), or (b) listing reported clinical symptoms being attributable to the reviewed imaging signs.

## 3 Results

Initial literature search found 1,226 articles in Web of Science^™^, 232 in PubMed and 9 in Google Scholar, which were narrowed down to 103 analyzed for article relevance depending on both title and abstract. The publication year ranged from 1993 to 2023. The full detail of the article selection process is documented in Table 1. Thirty-one articles including 1,484 dogs satisfied the inclusion criteria.

**Table 1:** Article selection process and final postulation.

Identification	Web of science^™^ (*n* = 1,226)	PubMed (*n* = 232)	Google scholar (*n* = 9)	“Occipital hypoplasia” OR “occipital dysplasia” OR “foramen magnum dysplasia” OR “incomplete ossification” OR “atlantoaxial overlapping” OR “syringohydromyelia” OR “syringomyelia” OR “Chiari-like malformation” AND “dog”
	Total (*n* = 103)			Full-text articles excluded by duplicity (*n* = 25)
Screening	Articles included for title and abstract screening (*n* = 88)			Full-text articles excluded for the following reasons (*n* = 57): Different focus Undefinable population Impaired correlation of imaging findings
Eligibility	Articles selected for full-text analysis (*n* = 31)			Full-text articles included with reasons based on CASP (*n* = 31)
Included	Articles included for qualitative analysis in this systematic minireview (*n* = 31)			Conclusion Only 3/249 dogs (1%) exhibited coexistence of OH, SM and AO. OH occurrence was reported in all dog groups, whereas SM was limited to small dogs, and AO was limited to small brachycephalic dogs. Five dogs had AO and clinical symptoms. AO could be a normal variation in small brachycephalic dogs. In the absence of SM, its clinical relevance should be carefully interpreted.
	Discussion			

### 3.1 Prevalence of OH, SM, and AO

The following small brachycephalic breeds were reported: Affenpinscher, Bichon Frise, Boston Terrier, Brussels Griffon, Cavalier King Charles Spaniel, Chihuahua, Japanese Chin, Maltese, Papillon, Pekingese, Pomeranian, Pug, Shih Tzu, Toy Poodle, and Yorkshire Terrier. Small non-brachycephalic breeds included Cocker Spaniel, Jack Russel Terrier, Miniature Dachshund, and West Highland White Terrier. Large brachycephalic breeds included Boxer and Staffordshire Bullterrier. Large non-brachycephalic breeds included Bull Terrier, Border Collie, Golden Retriever, Labrador Retriever, and Springer Spaniel.

Table 2 displays the breed group distributions for OH, SM, and AO. For articles focusing on descriptions of OH, SM, and AO, 249 dogs had at least one of these conditions, and 3 of these 249 dogs (1%) had coexistence of all three conditions (Supplementary Table S1). All 249 dogs were small brachycephalic dogs. The other combinations were distributed as follows: OH occurred in 24% (*n* = 59), SM in 4% (*n* = 9), AO in 7% (*n* = 18), OH and SM in 61% (*n* = 153), and SM and AO in 3% (*n* = 7). No dogs were reported to have coexisting OH and AO.

**Table 2:** Coexistence of occipital hypoplasia (OH), syringomyelia (SM) and atlanto-occipital overlapping (AO) in dogs reported between 1993 and 2023.

Group	OH	SM	AO	Total
Brachycephalic small breed	552	574	100	1,226
Non-brachycephalic small breed	19	2	0	21
Brachycephalic large breed	11	0	0	11
Non-brachycephalic large breed	11	6	0	17
Total	593	582	100	1,275

Because reports varied in the level of detail of the information, such as a condition not being found or not investigated, individual dogs were further classified into subgroups. OH and SM were concurrently reported in 217 dogs, with 156 (72%) showing imaging features of both entities, 59 (27%) showing only OH, and 2 (1%) showing only SM. Six dogs had OH and AO, with 3 (50%) showing either imaging signs of both or AO only. Thirty-seven dogs had SM and AO, with 10 (27%) showing both, 9 showing SM only (24%), and 18 showing AO only (49%). Six dogs had all three imaging findings together; 3 (50%) showed concurrent imaging findings of all three, 2 showed SM and AO (33%) and 1 (17%) showed only AO.

For all 1,484 dogs, we identified 1,275 individuals showing OH, SM, and AO in 552/19/11/11, 574/2/0/6, and 100/0/0/0 small brachycephalic, small non-brachycephalic, large brachycephalic, and large non-brachycephalic breeds, respectively (Table 2). Of the small brachycephalic dogs, the percentages of affected animals were 40% for OH (*p* < 0.01), 42% for SM (*p* < 0.01) and 7% for AO (*p* = 0.033).

Four studies of AO concurrently reported the following imaging findings: an abnormally large foramen magnum, cerebellar involvement, medullary kinking, atlantoaxial instability, dens abnormalities, and syringohydromyelia (16, 17, 19). In the 100 dogs, reported to be affected with AO, additional imaging findings described were medullary kinking (*n* = 95), atlantoaxial subluxation/instability (*n* = 67), occipital dysplasia (*n* = 67) hydrocephalus (*n* = 42), ventriculomegaly (*n* = 24), compressive lesions of the spinal cord (*n* = 39), arachnoid diverticula (*n* = 5), intervertebral disk protrusion/extrusion (*n* = 2), dens hypoplasia (*n* = 1), and central canal dilatation (*n* = 15).

### 3.2 Reported clinical symptoms

Twenty-three studies reported clinical symptoms. Thirty-eight clinical symptoms were described, including hyperesthesia/pain (*n* = 95), gait abnormalities (*n* = 60), decreased postural reactions (*n* = 33), neck and/or shoulder scratching (*n* = 35), proprioceptive deficits (*n* = 24), seizures (*n* = 22), decreased head reflexes (*n* = 21), and non-specific neurologic symptoms (*n* = 31). Other symptoms were mentioned in lower frequencies (*n* = ≤10) or only mentioned but not quantified. Supplementary Table S2 summarizes most frequently reported clinical symptoms in dogs affected with OH, SM, or AO. A total of 132 reported clinical symptoms were attributable to OH, SM, or AO. The table documents that the number of dogs having AO and clinical symptoms is low (*n* = 5).

## 4 Discussion

### 4.1 Principal findings

In this minireview, 249 dogs were identified as having OH, SM or AO, and all were small and brachycephalic (Supplementary Table S1). OH, SM and AO coexisted in 3/249 dogs (1%) (6, 22, 23). OH and SM were concurrent imaging findings in 153 dogs (61%). OH (Chiari-like malformation) is known to be associated with SM in dogs, where the skull is too small for the brain (24, 25). Dogs affected, most reported Cavalier King Charles Spaniels, show neck pain, scratching at the neck or shoulder, facial rubbing behavior, and vocalization among other clinical signs (26). Surgical decompression is indicated in severe cases (syrinx ≥ 3 mm diameter on transverse T2 MR images) and resulted, combined with medical therapy, in long-term improvement in most patients (27). Eighteen dogs (7%) exhibited AO as a single imaging finding. AO might be limited to small brachycephalic dog breeds owing to the typical head shape and geometry of the craniocervical junction. The short cranial base together with the caudodorsally orientated dome-shaped head (13) and consecutively cranioconvex-shaped foramen magnum lead to a geometrically well-covered atlas that might be wrongly interpreted on a median plane to be in the foramen magnum. Other than congenital causes (4), the discrepancy between the volume (or pressure) of the soft tissues and bones may lead to pressure atrophy of the occipital bone (resulting in OH or occipital dysplasia) and pressure atrophy of the dorsal arch of the atlas (paper-thin, no medullary canal) (28). The bone interacts with variable pressure conditions and reacts to them, which is described by the Wolff transformation law (29). The functional adaptation of the morphology of the head (dorsorotation of maxillary incisors, obstruction of the nasal cavity) (30) and atlas (highly oval-shaped and missing medullary cavity in the dorsal arch) (28) could also be a consequence of this local pressure increase.

### 4.2 Reevaluation of AO: is AO a key or a buried finding?

In this minireview, 38 clinical symptoms were described. Key (or major) findings significantly affect the odds of a specific diagnosis (31). Since 2009 (20), AO has been reported to be a key finding that influences the clinical signs and therapeutic outcome of dogs. Hence, surgical management is recommended (7). To date, AO has been surgically stabilized in two dogs; both were diagnosed with AO, but corresponding images documented coexisting severe atlantoaxial instability (32) or herniated cerebellar matter (19). A study of 41 dogs diagnosed with atlantoaxial instability and treated revealed that AO was a coexisting condition in 12 dogs. All dogs were surgically treated; however, presence or absence of AO did not affect the outcome (33). Conversely, buried findings are incidental (34) and not clinically significant. Apart from the original description, we did not identify further evidence that AO by itself is corresponding to clinical signs. However, in seven reported dogs AO and SM coexist (35, 36). Hence, whether AO represents normal variation in small brachycephalic dogs or is a key finding responsible for clinical signs and needs to be treated is unclear. Prospective observational clinical studies are needed to clarify the clinical relevance of AO.

### 4.3 Limitations

Limitations of this systematic review included the large variation of head morphologies among dog breeds and difficulties in correlating reported imaging findings and symptoms to individual dogs.

## 5 Conclusion

OH, SM and AO are more likely to affect small brachycephalic dogs than other types of dogs, possibly owing to the geometry of the craniocervical junction. Problems might arise from increased dorsorotation, which is associated with respiratory tract malformations (37) but also affects the craniocervical junction. Hence, AO alone might not lead to SM. We recommend classifying AO as a normal variation in small brachycephalic dogs rather than as a pathological finding that needs to be treated. In individual dogs, readers should carefully interpret the clinical relevance of OH or AO in the absence of SM.

## Author contributions

LW: Conceptualization, Data curation, Formal analysis, Investigation, Methodology, Project administration, Resources, Software, Supervision, Validation, Visualization, Writing – original draft, Writing – review & editing. SK: Conceptualization, Data curation, Formal analysis, Funding acquisition, Investigation, Methodology, Project administration, Resources, Software, Supervision, Validation, Visualization, Writing – original draft, Writing – review & editing.
