# Supplementary material for: Systematic minireview of the craniocervical junction in dogs with and without brachycephaly
Source: Front Vet Sci. 2024 May 31;11:1416670. doi: 10.3389/fvets.2024.1416670 (PMC11177753; doi:10.3389/fvets.2024.1416670)
Supplement: Supplementary file 1 [file Data_Sheet_1.docx]

**Supplementary Material**

**Table 3.** Summary of dog groups, occipital hypoplasia (OH), syringomyelia (SM), atlanto-occipital overlapping (AO) and other imaging findings in dogs reported between 1993 and 2023. Number of dogs, where at least two reviewed imaging signs were attributable, is listed in the column titled tagged.

|  |  |  |  | **Small breed** | | **Large breed** | | **Reviewed imaging findings** | | | | **Other imaging findings** |
| --- | --- | --- | --- | --- | --- | --- | --- | --- | --- | --- | --- | --- |
| **No.** | **Ref.** | **Year** | **Study design** | **B** | **NB** | **B** | **NB** | **OH** | **SM** | **AO** | **Tagged** |  |
| 1 | (38) | 1993 | case report^#^ | 1 |  |  |  | 1 |  |  |  | Hydrocephalus, occipital dysplasia |
| 2 | (5) | 1996 | case report^#^ | 2 |  |  |  |  | 2 |  |  | Occipital dysplasia, ventriculomegaly |
| 3 | (39) | 2000 | case report^#^ | 2 |  |  |  |  | 1 |  |  | C7 subluxation, dilated lateral ventricles, hydrocephalus |
|  |  |  |  |  | 2 |  |  |  | 2 |  |  | Atlantoaxial subluxation, hypoplasia, dilated third and fourth ventricle, dilated subarachnoid space |
|  |  |  |  |  |  |  | 1 |  | 1 |  |  | Hydrocephalus, ventriculomegaly |
| 4 | (15) | 2000 | retrospective^#^ | 7 |  |  |  | 7 | 7 |  | 7 | Occipital dysplasia, hydrocephalus |
| 5 | (40) | 2003 | retrospective^#^ | 40 |  |  |  | 40 | 26 |  | 40 | Hydrocephalus |
| 6 | (6) | 2004 | retrospective^#^ | 27 |  |  |  | 27 | * |  |  |  |
|  |  |  |  |  | 3 |  |  | 3 |  |  |  |  |
| 7 | (41) | 2004 | retrospective^#^ | 50 |  |  |  | 50 | 50 |  | 50 | Hydrocephalus |
| 8 | (42) | 2006 | case report^#^ | 2 |  |  |  | 2 | 2 |  | 2 | Occipital dysplasia |
| 9 | (43) | 2008 | prospective | 16 |  |  |  | 16 | 7 |  |  |  |
| 10 | (22) | 2008 | case report^#^ | 1 |  |  |  | 1 | 1 |  | 1 | Occipital dysplasia |
| 11 | (20) | 2009 | case report^#^ | 4 |  |  |  | 1 | 3 | 4 | 4 | Atlantoaxial subluxation, dens hypoplasia, medullary kinking, occipital dysplasia, ventriculomegaly |
| 12 | (44) | 2008 | prospective^#^ | 35 |  |  |  | 25 | 6 |  | 25 |  |
| 13 | (18) | 2009 | prospective^#^ | 64 |  |  |  | 59 | 27 |  |  | »Dorsal compressive lesion (C1/2)«, medullary kinking, occipital dysplasia, ventriculomegaly |
| 14 | (19) | 2009 | case report^#^ | 1 |  |  |  | 1 | 1 | 1 | 1 |  |
| 15 | (45) | 2010 | case report^#^ | 1 |  |  |  | 1 | 1 |  | 1 | Hydrocephalus, intervertebral disc protrusion/extrusion, occipital dysplasia |
| 16 | (4) | 2013 | retrospective^#^ | 67 |  |  |  | 67 | 52 |  | 67 | Brainstem infarct, meningoencephalomyelitis, oligoastrocytoma, otitis media |
| 17 | (46) | 2014 | prospective^#^ | 84 |  |  |  | 55 | 44 |  |  |  |
| 18 | (47) | 2015 | retrospective^#^ |  |  |  | 5 |  | 5 |  |  | Arachnoid diverticle (in fourth ventricle) with secondary obstructive hydrocephalus |
| 19 | (23) | 2015 | retrospective | 14 |  |  |  | 14 |  |  |  |  |
|  |  |  |  |  | 23 |  |  | 16 |  |  |  |  |
|  |  |  |  |  |  | 12 |  | 10 |  |  |  |  |
|  |  |  |  |  |  |  | 47 | 11 |  |  |  |  |
| 20 | (32) | 2016 | case report^#^ | 1 |  |  |  |  |  | 1 |  | Atlantoaxial instability, occipital dysplasia |
| 21 | (48) | 2016 | retrospective | 19 |  |  |  | 19 | 8 |  | 19 | Hydrocephalus |
| 22 | (49) | 2017 | prospective^#^ | 27 |  |  |  | 19 |  |  |  |  |
|  |  |  |  |  |  | 3 |  | 1 |  |  |  |  |
| 23 | (50) | 2017 | case report^#^ | 1 |  |  |  | 1 | 1 | 1 | 1 | Atlantoaxial subluxation/instability |
| 24 | (51) | 2017 | prospective^#^ | 53 |  |  |  | 53 | 20 | 35 |  | Central canal dilatation, medullary kinking |
| 25 | (35) | 2018 | retrospective | 96 |  |  |  | 61 |  |  |  |  |
|  |  |  |  | 461 |  |  |  |  | 274 |  |  |  |
| 26 | (36) | 2019 | retrospective | 55 |  |  |  |  | 14 | 22 | 31 | Atlantoaxial instability, »dorsal compression (of the spinal cord)« |
|  |  |  |  |  | 2 |  |  |  |  |  |  |  |
| 27 | (52) | 2019 | retrospective | 122 |  |  |  |  |  | 36 |  | Atlantoaxial instability |
| 28 | (53) | 2019 | retrospective | 7 |  |  |  |  | 7 |  |  |  |
| 29 | (54) | 2021 | prospective^#^ | 50 |  |  |  |  | 20 |  |  | Dilated fourth ventricle |
| 30 | (55) | 2023 | prospective | 30 |  |  |  |  |  |  |  |  |
|  |  |  |  |  |  |  | 14 |  |  |  |  |  |
| 31 | (56) | 2023 | retrospective^#^ | 32 |  |  |  | 32 | * | * |  | Syringobulbia |
| Total |  |  |  | 1372 | 30 | 15 | 67 | 593 | 582 | 100 | 249 |  |

AO, atlanto-occipital overlapping; B, brachycephalic; NB, non-brachycephalic; OH, occipital hypoplasia; SM, syringomyelia; *mentioned in the citation, but not considered, because breeds/grouping was ambiguous; ^#^studies listing clinical signs.

**Table 4.** Summary of most frequently reported clinical symptoms in dogs affected with occipital hypoplasia (OH), syringomyelia (SM), atlanto-occipital overlapping (AO). A total of 132 reported clinical symptoms were attributable to OH, SM or AO.

| **Imaging sign** | **hyperaesthesia/pain** | **gait**  **abnormalities** | **decreased**  **postural reactions** | **neck/shoulder scratching** | **proprioception deficits** | **seizures** | **decreased head reflexes** | **Total** |
| --- | --- | --- | --- | --- | --- | --- | --- | --- |
| **OH** | 2* | 17* | 1 | 0 | 0 | 17* | 0 | 37 |
| **SM** | 13* | 14* | 4 | 0 | 2 | 1 | 0 | 34 |
| **AO** | 2 | 1 | 0 | 0 | 1 | 0 | 1 | 5 |
| **OH +SM** | 14 | 4 | 0 | 20 | 6 | 0 | 0 | 44 |
| **AO + OH** | 0 | 0 | 0 | 0 | 0 | 0 | 0 | 0 |
| **AO + SM** | 2 | 2 | 0 | 0 | 2 | 0 | 0 | 6 |
| **AO + OH + SM** | 2 | 3 | 0 | 0 | 1 | 0 | 0 | 6 |
| **Total** | 35 | 41 | 5 | 20 | 12 | 18 | 1 | **132** |

*Listed but probability of error corresponds to +1, because breeds/grouping was ambiguous.
